# DNA Sequencing from Subcritical Concentration of Cell-Free DNA Extracted from Electrowetting-on-Dielectric Platform

**DOI:** 10.3390/mi13040507

**Published:** 2022-03-25

**Authors:** Anand Baby Alias, Hong-Yuan Huang, Yi-Wen Wang, Kai-Ti Lin, Pei-Jhen Lu, Tzu-Hui Wu, Pei-Shin Jiang, Chien-An Chen, Da-Jeng Yao

**Affiliations:** 1 Institute of NanoEngineering and MicroSystems, National Tsing Hua University, Hsinchu 30013, Taiwan; alias.anand@gmail.com; 2 Department of Obstetrics and Gynecology, Chang Gung Memorial Hospital, Taoyuan 33305, Taiwan; iwen0711@gmail.com (Y.-W.W.); t2003456@gmail.com (P.-J.L.); 3 Department of Obstetrics and Gynecology, College of Medicine, Chang Gung University, Taoyuan 33305, Taiwan; 4 College of Life Science, National Tsing Hua University, Hsinchu 30013, Taiwan; 5 Biomedical Technology and Device Research Labs, Industrial Technology Research Institute (ITRI), Hsinchu 31057, Taiwan; hui0814@itri.org.tw (T.-H.W.); pspeggy@itri.org.tw (P.-S.J.); cachen@itri.org.tw (C.-A.C.)

**Keywords:** cf-DNA extraction, EWOD, modified nested PCR, DNA sequencing, BLAST

## Abstract

Electro-Wetting-On-Dielectric (EWOD) based digital operations have demonstrated outstanding potential in actuating and manipulating liquid droplets. Here, we adapted the EWOD for extracting femtogram quantities of cell-free DNA (cf-DNA) from 1 μL of KSOM mouse embryo culture medium. Our group extracted the femtogram quantity of cf-DNA from 1 μL of mouse embryo culture medium in our previous work. Here, we initially explain a modification from our previous extraction protocol, which improves the extraction percentage to 36.74%. Though the modified extraction protocol improves the extraction percentage from our previously reported work, the quantity is still in the femtogram range. The cf-DNA in femtogram quantity is in subcritical/subthreshold concentration for any further analysis, such as sequencing. To the best of our knowledge, we need a minimum of picogram/nanogram DNA quantities for further analysis. We demonstrated a ground-breaking mechanism of this subcritical concentration of cf-DNA amplification to the nanogram range and performed DNA sequencing. Basic Local Alignment Search Tool (BLAST) is used as a sequence similarity search program to confirm the identity percentage between query and subject. More than 97% of nucleotide identities between query and subject sequences have been obtained from the sequencing result. Hence, we can use the methodology to amplify the subcritical concentration of extracted DNA for further analytics. Moreover, as we extract the cf-DNA from the embryo culture medium, the natural growth of the embryo has not been disrupted. This entire mechanism will pave a new path towards the lab-on-a-chip (LOC) concept.

## 1. Introduction

Genetic analysis procedures include three major steps: (1) DNA extraction from raw biological samples, (2) sequence amplification with the real-time polymerase chain reaction (PCR) and (3) separation and selection of DNA for testing [1]. Here, we demonstrate the first two steps in genetic analysis as explained above, though the extracted DNA is in subcritical concentration to perform sequencing. 

In the replication of DNA and the shortening of telomeres, DNA nucleotides act as magnets [2]. Magnetic beads (MB) can be used as labels to concentrate or separate biological targets from a sample, such as cells, proteins, and DNA/RNA [3]. 

Various cf-DNA extraction methods are explained in [4]. In all conventional ways of DNA extraction, a milliliter quantity of sample is required. Thus, the conventional molecular biology method requires bio-reagents in a large-scale to perform DNA extraction. Digital microfluidics (DMF) maintain low sample quantity (microliter range), high throughput, repeatability and lower operational costs by reducing reagent quantity requirements [5]. EWOD is a DMF methodology that was developed as a mechanism for implementing LOC systems. A microfluidic function can be reduced to a collection of basic operations by manipulating droplets of microscale volumes along electrode arrays [6]. Microfluidic operations with EWOD actuation offer precise droplet actuation, reduced risk of contamination, reduced reagent volume, reduced reaction time, and improved reagent mixing efficiency [1]. Active management of produced droplets can greatly speed up mixing in electrowetting systems [7]. The aqueous droplets can transmit over the EWOD surface thanks to a series of potentials applied to neighboring electrodes on an array [8]. To implement the extraction, we employed the ‘DropBot Model: DB3–120’ (Sci-bots, Kitchener, ON, Canada) as an EWOD platform [9].

In our previous work, our group extracted the femtogram quantity of cf-DNA from 1 μL of mouse embryo culture medium [1]. The quantity is not enough for any further analysis. To our best understanding, we need a minimum of nanogram DNA quantity for further analysis, such as sequencing. 

DNA sequencing is any chemical, enzymatic or technological process to determine the linear order of nucleotides in DNA. DNA sequencing has extremely changed the nature of biomedical research and the medical field [10]. A minimum of nanogram range DNA is required for sequencing [11]. 

Although the end-point PCR uses the same thermal cycler and temperature cycling conditions [12], the exponential nature of DNA amplification is prone to burdening experimental data with significant standard error due to inherent variations in amplification efficiency from tube to tube [13]. A standard curve (calibration curve) is a sort of graphical analysis used as a quantitative research technique in which several samples with known qualities are measured and graphed, and the same properties are deduced for unknown samples via graph interpolation [14]. To perform quantitative analysis of unknown cf-DNA samples, we employed known amounts of mouse g-DNA for standard curve analysis.

Nested PCR is a secondary approach that uses the PCR principle. Due to two rounds of amplification, nested PCR provides greater sensitivity and specificity. Nested PCR is better for detecting samples with the lowest virus load [15,16]. In nested endpoint PCR assay, two rounds of conventional PCR and gel electrophoresis are incorporated [17]. Though nested primer PCRs are widely employed for detection, examples of applying nested primer PCR to quantitative applications are absent [18].

Basic Local Alignment Search Tool (BLAST) is a sequence similarity search program that can be used via a web interface for comparing a user’s query to a database of sequences [19,20,21].

In this paper, we report the DNA sequencing of subcritical concentration of cf-DNA samples. A modification in the extraction of cf-DNA from mouse embryo culture medium from [1] makes an improved extraction percentage. The implemented PCR methodology amplifies the final PCR product from a subcritical femtogram concentration of cf-DNA to the nanogram level, allowing DNA sequencing to be performed. As a result, the EWOD extracted subcritical concentration of cf-DNA from a microscale quantity of mouse embryo culture media can be used for sequencing or other analyses. 

## 2. Materials and Methods

cf-DNA Extraction: We developed an MB based DNA extraction protocol in the EWOD system. Our methodology can extract cf-DNA from a mouse embryo culture medium at a small concentration. In the EWOD platform, only a microliter quantity of bio-buffers is utilized for the extraction procedure. The magnetic property of DNA enables them to bind with the magnetic beads. Elution buffer is used to detach the cf-DNA from the magnetic beads. The mouse embryo culture medium is obtained from Chang Gung Memorial Hospital (CGMH), Linkou, Taiwan, and the bio-buffers are obtained from Industrial Technology Research Institute (ITRI), Hsinchu, Taiwan.

The mouse embryo culture medium is kept at −80 °C. The bio-buffers for cf-DNA include lysis buffer, carrier-RNA, proteinase K, wash buffer-1, wash buffer-2, elution buffer along with magnetic beads. Table S1 represents the storage temperature required for each bio-buffers.

**Figure 1:**
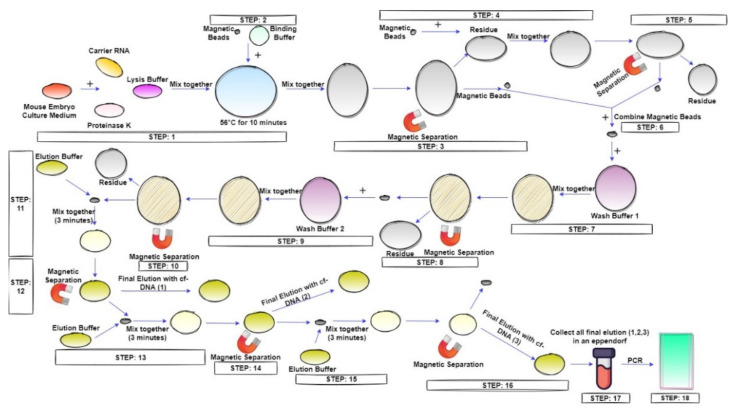
The typical way of magnetic bead based cf-DNA extraction. Step 1: KSOM mouse embryo culture medium is mixed with Proteinase K, carrier RNA, and lysis buffer. The mixture is heated at 56 °C for 10 min. Step 2: Add binding buffer and magnetic beads to the heated mixture. Perform vortex of MB at 99 rpm for 1 min before adding to the mixture. Step 3: Use an external magnet to remove the supernatant (residue) from MB. Step 4: Add more MB to the supernatant. This step is to collect the unbounded cf-DNA (if any) in the supernatant. Step 5: Use an external magnet to remove the supernatant. From MB. Step 6: Combine the magnetic beads (from step 3 and step 5). Step 7: Add wash buffer-1 to the MB. Step 8: Use an external magnet to remove the supernatant from MB. Step 9: Add wash buffer-2 to the MB. Step 10: Use an external magnet to remove the supernatant from MB. Step 11: Add elution buffer to the MB. Mix for 3 min. Step 12: Use an external magnet to remove the supernatant from MB. Step 13: Add more elution buffer to the MB. This step enables the collection of more cf-DNA binds with the MB after step-11 and step-12. Step 14: Use an external magnet to remove the supernatant from MB. Step 15: Add more elution buffer to the MB. This step further enables the collection of more cf-DNA which binds on the MB even after step-13 and step-14. Step 16: Use an external magnet to remove the supernatant from MB. Step 17: Collect all the elution buffer in an Eppendorf for PCR amplification.

The typical way of cf-DNA extraction is demonstrated in Figure 1. The entire extraction procedure is carried out in seventeen steps.

We used the EWOD platform of DropBot–SCI bots for the experiment. 

### 2.1. Calculation of Extraction Percentage by g-DNA

The quantitative PCR (q-PCR) method is used for the quantitative analysis of the EWOD extracted cf-DNA. We used g-DNA (of known concentration; 100 ng) from the mouse tail to calculate our protocol’s extraction percentage. The g-DNA has undergone the extraction steps and we performed q-PCR with the final elution buffer. The equation to find the percentage of extraction is E (percentage) = [1/2 [C_t_(B) − C_t_(A)]] × 100 in which E (percentage) = extraction percentage, C_t_(B) = cycle threshold value of template, C_t_(A) = cycle threshold value of control (100 ng of g-DNA) [1]. 

### 2.2. EWOD cf-DNA Extraction from Mouse Embryo Culture Medium

The EWOD chip [22] has a top plate and a bottom plate; the gap between these plates is 180 µm; the dimensions of the electrode are 2.25 mm × 2.25 mm. Each transport electrode can hold 1 µL of bio-buffer. However, it strictly depends on the number of transportation electrodes used for activation (ON/OFF). An amount of 1 µL is generated when we activate one transportation electrode along with the generation electrode. 

The video in the supplementary information shows the cf-DNA extraction in the EWOD platform. We have adapted the extraction protocol explained in Figure 1 to EWOD for the cf-DNA extraction from 1 μL mouse embryo culture medium of 2.5 days (E2.5) and 3.5 days (E3.5). As the quantity of EWOD extracted cf-DNA is in the femtogram range, we cannot utilize the sample for any further genomic analytics.

### 2.3. Amplification of Subcritical Concentration of EWOD Extracted cf-DNA

The quantity of cf-DNA in the mouse embryo culture medium is in the femtogram range. Further analysis of cf-DNA is impossible in the femtogram range. For overcoming the current limitation, we have developed a modified form of nested PCR. Here, the resultant sample of first-time PCR was considered as the template to run second-time PCR with the same primer. We need to freshly prepare the master-mix, primer forward, primer reverse, and DD water for the second-time PCR. In place of the template, we need to add the resultant sample of first-round PCR.

**Figure 2:**
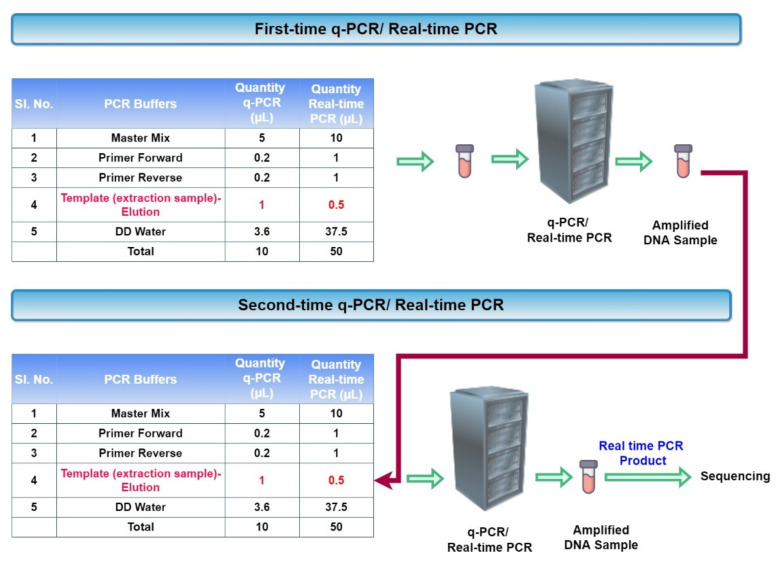
Modified nested q-PCR and real-time PCR protocol.

The modified nested PCR protocol for both q-PCR and real-time PCR is given in Figure 2. In q-PCR, for the quantitative analysis, a fluorescent master-mix is used. Hence, we cannot use the final sample result for any further analysis as sequencing. So, we performed the modified nested PCR protocol in real-time PCR.

The sequence of Primer is given below:

Primer Forward: GTCTCATCACAAACATTCCCAC

Primer Reverse: GTTGGGGTAATGAATGAGGC

The primer (Mus musculus voucher MA364 mitochondrion–Mus musculus) is for the amplification of mt-DNA

The resultant sample of the second-time traditional PCR is further given for DNA sequencing. 

### 2.4. DNA Sequencing and BLAST Sequence Similarity Validation

The second-time PCR product was subjected to sequencing and compared to the result of the positive control (g-DNA of 100 ng) in BLAST to check the validity of amplified cf-DNA which is in the nanogram range. We have performed the sequencing with both forward and reverse primer.

BLAST Sequence Similarity Validation of E2.5 and E3.5 Samples

The DNA sequence shown in Table 1 is the trimmed sequence after the deletion of noisy initial and final nucleotides. Usually, we need to ignore the noisy initial and final nucleotides. Here we can observe a matched sequencing result between E2.5 and E3.5 with forward primer (green), E2.5 and E3.5 with reverse primers (blue). Table S2 shows the raw sequencing data of E2.5 and E3.5 along with the subject query (g-DNA).

## 3. Results

### 3.1. Recovery Rate of g-DNA in Conventional and EWOD Way

**Figure 3:**
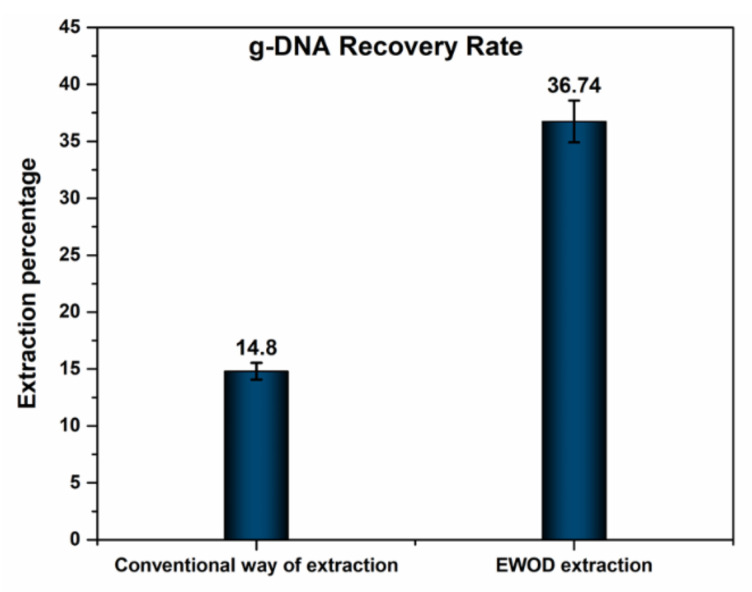
Extraction percentage (recovery rate) of g-DNA in conventional and EWOD way.

The conventional extraction gives an extraction of 14.8% [1], whereas 36.74% on the EWOD platform as given in Figure 3. Hence an improvement in EWOD extraction has been achieved. An improvement in extraction percentage from our previously reported work has been achieved. For getting the best extraction result, at step 2, vibrate/vortex the magnetic beads for 1 min at 99 rpm. This makes the magnetic beads spread all-over the sample mixture to obtain better cf-DNA bonding. During the final elution stage, we need to continuously mix the sample (MB elution buffer) via electrode movements for 3 min.

### 3.2. Extraction Quantity of cf-DNA after First and Second-Time q-PCR

**Figure 4:**
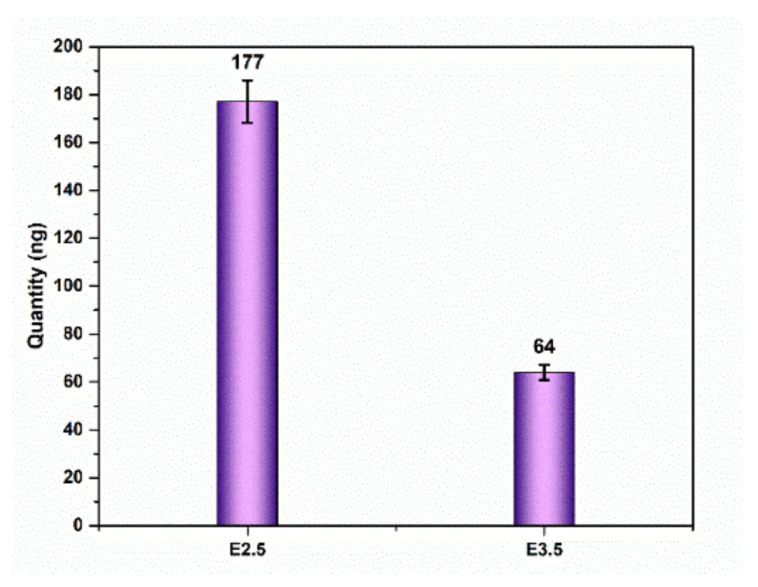
cf-DNA quantity after second-time q-PCR.

Despite the fact that the modified extraction protocol improves on our earlier work in terms of extraction percentage, the quantity remains in the femtogram range after first-time q-PCR. After the second-time q-PCR, the cf-DNA quantity amplified from the subcritical femtogram range to the nanogram range as given in Figure 4. The nanogram quantity of cf-DNA is enough for further analysis.

**Figure 5:**
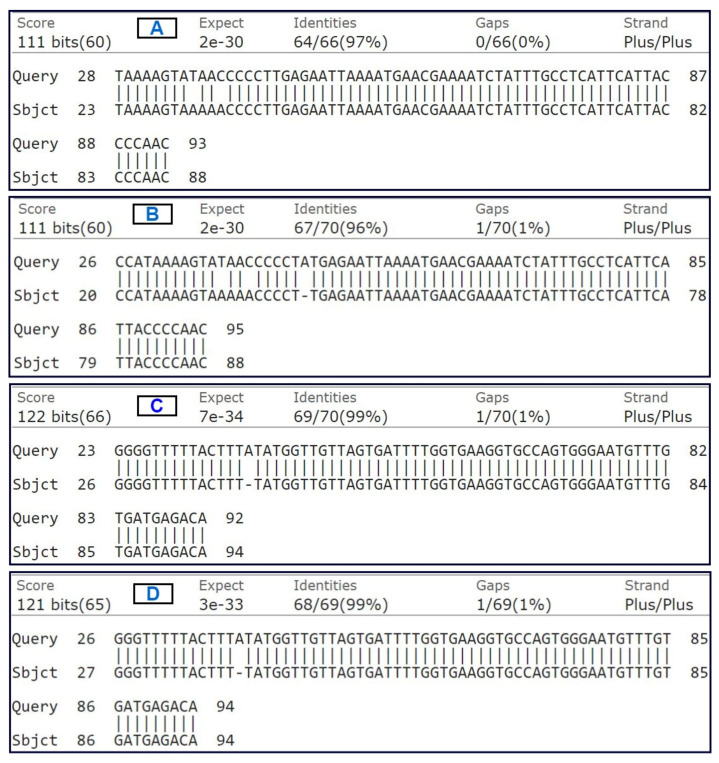
BLAST sequence similarity validation. (**A**) Query sequence: second-time PCR product of E2.5 with forward primer & subject sequence: g-DNA (positive control) sequencing with forward primer; (**B**) Query sequence: second-time PCR product of E3.5 with forward primer & subject sequence: g-DNA (positive control) sequencing with forward primer; (**C**) Query sequence: second-time PCR product of E2.5 with reverse primer & subject sequence: g-DNA (positive control) sequencing with reverse primer; (**D**) Query sequence: second-time PCR product of E3.5 with reverse primer & subject sequence: g-DNA (positive control) sequencing with reverse primer.

Figure 5 represents the BLAST identity percentage of samples mentioned in Table S2. For the sequence similarity validation, the raw sequence data of the second-time PCR product of E2.5 and E3.5 (which is in the nanogram range) is considered as the query sequence whereas the raw sequence data of mouse g-DNA of 100 ng sample is considered as the subject sequence. The result shows that the second-time amplified cf-DNA samples (in nanogram range) have 96 to 99 percent of identity with the mouse g-DNA (positive control). The result validates our EWOD extraction protocol and the modified nested-PCR procedure. Hence, by following the protocol, the subcritical concentration cf-DNA extracted from 1 μL of mouse embryo culture medium can be utilized for sequencing or any further analysis as per requirement.

## 4. Discussion

For the experiment, we employed the DropBot–SCI bots EWOD platform. Photolithography was employed to form bottom-plates with chromium electrodes, while Teflon-AF was used to cover ITO-coated glass substrates as top-plates [9]. A typical way of magnetic bead based cf-DNA extraction has been put forward. Before mixing with the sample mixture, the magnetic beads initially need to vortex/vibrate at 99 rpm for 1 min. This allows the magnetic beads to spread all-over the sample mixture to obtain better cf-DNA binding. The electrode mixing of elution buffer with the MB for 3 min enhances the extraction percentage. With this modified extraction protocol, EWOD extraction of cf-DNA from the micro-scale quantity of mouse embryo culture medium is carried out. As we used the embryo medium culture for DNA extraction, there was no harm to the embryo growth, and cryopreservation was not required. As the quantity of cf-DNA is in the femtogram, we cannot perform any further analytical studies, such as sequencing. To the best of our knowledge, a minimum nanogram quantity of cf-DNA is required for performing sequencing. Hence, we implemented a modified nested PCR method to amplify cf-DNA from femtogram to nanogram quantity. The amplified DNA product of first-time PCR is used as the template in second-time PCR with the same primer set. The obtained nanogram range of cf-DNA was sequenced and sequence similarity was determined by BLAST to determine the identity percentage between the query and subject. The sequence similarity proves that the proposed cf-DNA extraction and PCR methods can be utilized to amplify subcritical (femtogram) cf-DNA concentration to nanogram range which can be implemented in LOC based clinical genetic DNA testing.

## 5. Conclusions

EWOD based digital operations have been implemented for the extraction of cf-DNA from mouse embryo culture medium. A typical way of magnetic bead based cf-DNA extraction has been demonstrated and successfully implemented. Our EWOD cf-DNA extraction mechanism extracts a femtogram quantity of cf-DNA from E2.5 & E3.5 days of 1 μL of KSOM embryo culture medium. A minimum of picogram/nanogram DNA quantity is needed for further analysis, such as DNA sequencing. We demonstrated and successfully implemented a modified nested-PCR amplification method, where the amplified DNA product of first-time PCR is used as the template in second-time PCR with the same primer set. As a result, the final PCR product amplifies from subcritical femtogram concentration of cf-DNA to the nanogram range for performing DNA sequencing. The sequence similarity determination tool, BLAST, is used to confirm the identity percentage between query and subject. More than 97% of nucleotide identities have been obtained in the sequencing. Hence, the EWOD extracted cf-DNA from 1 μL of mouse embryo culture medium which is at subcritical concentrations can be utilized for sequencing or any further analysis as per requirements.

## Figures and Tables

**Table 1:** The sample used as query with their respective DNA sequence obtained.

Sample Type	DNA Sequence
Second-time PCR product of E2.5 with forward primer	TAAAAGTATAACCCCCTTGAGAATTAAAATGAACGAAAATCTATTTGCCTCATTCATTACCCCAAC
Second-time PCR product of E2.5 with reverse primer	GGGTTTTTACTTTATATGGTTGTTAGTGATTTTGGTGAAGGTGCCAGTGGGAATGTTTGTGATGAGACA
Second-time PCR product of E3.5 with forward primer	TAAAAGTATAACCCCCTATGAGAATTAAAATGAACGAAAATCTATTTGCCTCATTCATTACCCCAAC
Second-time PCR product of E3.5 with reverse primer	GGGTTTTTACTTTATATGGTTGTTAGTGATTTTGGTGAAGGTGCCAGTGGGAATGTTTGTGATGAGACA

## Supplementary Materials

The following supporting information can be downloaded at: https://www.mdpi.com/article/10.3390/mi13040507/s1, Table S1: Bio-buffers required for cf-DNA extraction and its storage temperature; Table S2: The query sequencing data with subject sequence; Video S1: cf-DNA extraction in EWOD platform.
