# Supplementary material for: DNA Sequencing from Subcritical Concentration of Cell-Free DNA Extracted from Electrowetting-on-Dielectric Platform
Source: Micromachines (Basel). 2022 Mar 25;13(4):507. doi: 10.3390/mi13040507 (PMC9031944; doi:10.3390/mi13040507)
Supplement: Supplementary file 1 [file micromachines-13-00507-s001.zip › micromachines-1649673 SI.pdf]

*DNA Sequencing from Subcritical Concentration of cf-DNA Extracted from EWOD*

# Supplementary Information:

The mouse embryo culture medium is kept at -80degree C. The bio-buffers for cf-DNA and their storage temperature are listed in table S1.

**Table S1: Bio-buffers required for cf-DNA extraction and its storage temperature**

| Sl. No. | Bio-Buffers | Storage Temperature (In Degree C) |
|---|---|---|
| 1 | Lysis Buffer | 6 |
| 2 | Carrier-RNA | -20 |
| 3 | Proteinase K | 6 |
| 4 | Magnetic Beads | 6 |
| 5 | Wash Buffer-1 | 6 |
| 6 | Wash Buffer-2 | 6 |
| 7 | Elution Buffer | 6 |

Table S2 shows the raw sequencing data of E2.5 and E3.5 along with the subject query. For the better understanding, the noisy regions are given in grey colour. After ignoring the grey region in Query Sequence, we can observe perfect match between E2.5 and E3.5 (forward) & E2.5 and E3.5 (reverse).

**Table S2: The query sequencing data with subject sequence**

| Sample Type | DNA Sequence |
|---|---|
| ***Query Sequence:*** Second-time PCR product of E2.5 with forward primer | TAAGAAGAGCGTGAGAAGTAGCACACTTAA AAGTATAACCCCCTTGAGAATTAAAATGAAC GAAAATCTATTTGCCTCATTCATTACCCCAAC AGA |
| ***Query Sequence:*** Second-time PCR product of E2.5 with reverse primer | TAAACCTGCACACACAGAGAAGGGGGTTTTT ACTTTATATGGTTGTTAGTGATTTTGGTGAAG GTGCCAGTGGGAATGTTTGTGATGAGACA |
| ***Query Sequence:*** Second-time PCR product of E3.5 with forward primer | GTAGCTAGCGTCTTTATGTGCTGTGCCATAAA AGTATAACCCCCTATGAGAATTAAAATGAAC GAAAATCTATTTGCCTCATTCATTACCCCAAC AAA |
| ***Query Sequence:*** Second-time PCR product of E3.5 with reverse primer | GTATGCATCACACACAACAGTAGTAGGGTTT TTACTTTATATGGTTGTTAGTGATTTTGGTGA AGGTGCCAGTGGGAATGTTTGTGATGAGACA |
| ***Subject sequence:*** g-DNA (Positive control) sequencing with forward primer | GTAGTATCACTCACTAACACCATAAAAGTAA AAACCCCTTGAGAATTAAAATGAACGAAAAT CTATTTGCCTCATTCATTACCCCAACCAA |
| ***Subject sequence:*** g-DNA (Positive control) sequencing with reverse primer | GTTAGTTACGTTCGTGTAGATCTCAGGGGTTT TTACTTTTATGGTTGTTAGTGATTTTGGTGAA GGTGCCAGTGGGAATGTTTGTGATGAGACA |
